# The Hyperferritinemic Syndrome: macrophage activation syndrome, Still’s disease, septic shock and catastrophic antiphospholipid syndrome

**DOI:** 10.1186/1741-7015-11-185

**Published:** 2013-08-22

**Authors:** Cristina Rosário, Gisele Zandman-Goddard, Esther G Meyron-Holtz, David P D’Cruz, Yehuda Shoenfeld

**Affiliations:** 1 Center for Autoimmune Diseases, Sheba Medical Center (affiliated with the Tel-Aviv University), Tel-Hashomer 52621, Israel; 2 Sackler Faculty of Medicine, Tel-Aviv University, Tel-Aviv, Israel; 3 Department of Medicine C, Wolfson Medical Center, Holon, Israel; 4 Department of Biotechnology and Food Engineering, Technion - Israel Institute of Technology, Haifa, Israel; 5 Louise Coote Lupus Unit, Guy’s and St Thomas’ Hospital, London, England, UK

**Keywords:** Hyperferritinemia, Macrophage activation syndrome (MAS), Adult onset Still’s disease (AOSD), Catastrophic antiphospholipid syndrome (cAPS), Septic shock

## Abstract

**Background:**

Over the last few years, accumulating data have implicated a role for ferritin as a signaling molecule and direct mediator of the immune system. Hyperferritinemia is associated with a multitude of clinical conditions and with worse prognosis in critically ill patients.

**Discussion:**

There are four uncommon medical conditions characterized by high levels of ferritin, namely the macrophage activation syndrome (MAS), adult onset Still’s disease (AOSD), catastrophic antiphospholipid syndrome (cAPS) and septic shock, that share a similar clinical and laboratory features, and also respond to similar treatments, suggesting a common pathogenic mechanism. Ferritin is known to be a pro-inflammatory mediator inducing expression of pro-inflammatory molecules, yet it has opposing actions as a pro-inflammatory and as an immunosuppressant. We propose that the exceptionally high ferritin levels observed in these uncommon clinical conditions are not just the product of the inflammation but rather may contribute to the development of a cytokine storm.

**Summary:**

Here we review and compare four clinical conditions and the role of ferritin as an immunomodulator. We would like to propose including these four conditions under a common syndrome entity termed “Hyperferritinemic Syndrome”.

## Background

For most clinicians dealing with inflammatory diseases, serum ferritin levels are a rather non-specific marker of the acute phase response, which is often ignored or not measured when the patient presents acutely. In some diseases, ferritin levels may be extremely high and, while not specific, these very high levels may be helpful diagnostically. Four uncommon immune mediated conditions may be associated with high ferritin levels: macrophage activation syndrome (MAS), adult onset Still’s disease (AOSD), catastrophic antiphospholipid syndrome (cAPS) and septic shock. These disorders share similar clinical and laboratory presentations and they also respond to similar treatments, suggesting that hyperferritinemia may be involved in a common pathogenic mechanism.

There is increasing evidence that circulating ferritin levels may not only reflect an acute phase response but may play a critical role in inflammation [1]. Its secretion is regulated by pro-inflammatory cytokines and ferritin has immunosuppressive effects possibly mediated by binding to its receptor [2]. Different mechanisms may inhibit the ferritin-mediated suppression of the immune cells, and in turn, this impaired immunosuppression may favor the loss of tolerance and the development of autoimmune diseases [2]. Moderate levels of hyperferritinemia are associated with autoimmune diseases, including systemic lupus erythematosus (SLE), rheumatoid arthritis (RA), multiple sclerosis (MS) [3-7] and antiphospholipid syndrome (APS) [8]. Although it is generally accepted that circulating ferritin levels may reflect an acute phase response, the explanation for why and how serum ferritin is elevated is unknown.

We hypothesize that the huge levels of ferritin seen in these four clinical conditions are not just a secondary product of the inflammatory process but rather they are part of the pathogenic mechanism. Therefore, we propose to include them under a single nomenclature: “The Hyperferritinemic Syndrome”.

### Ferritin

Ferritin is an iron-binding molecule that stores iron in a biologically available form for vital cellular processes while protecting proteins, lipids and DNA from the potential toxicity of this metal element. Ferritin plays a role in a large number of other conditions, including inflammatory, neurodegenerative and malignant diseases [9].

**Figure 1:**
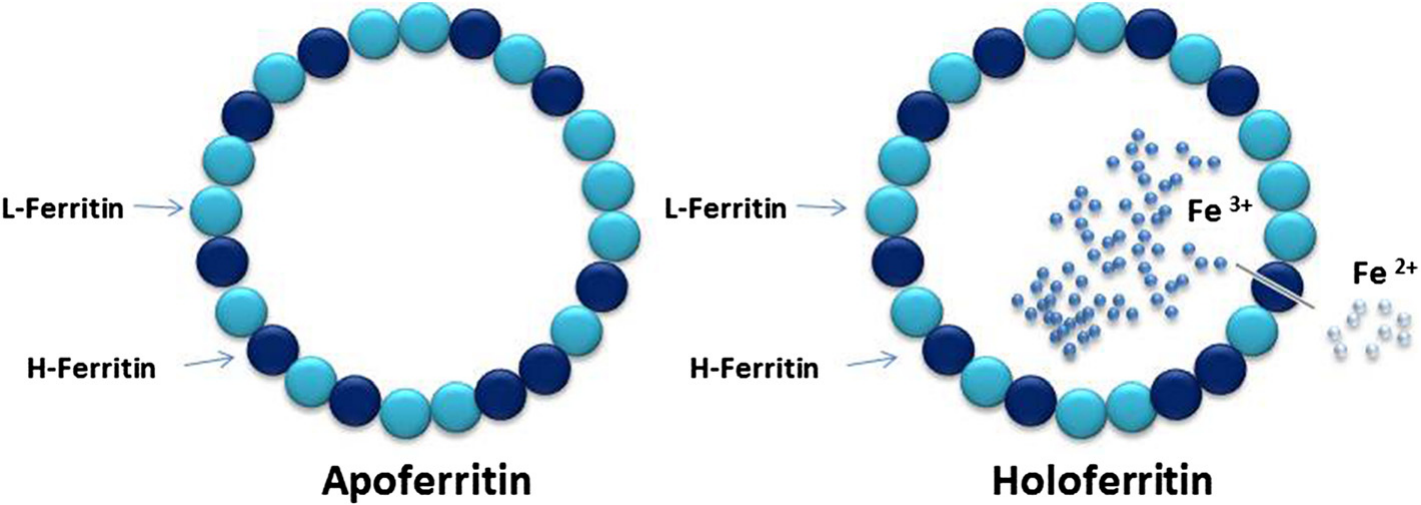
**Ferritin structure and function.** Ferritin is a major intracellular iron storage protein in all organisms, and its structural properties are largely conserved through species. Apoferritin refers to the iron-free form of the protein; the iron-containing form is termed holoferritin or simply ferritin. Each apoferritin shell comprises 24 subunits of two kinds: a H-subunit and a L-subunit. Depending on the tissue type and physiologic status of the cell, the ratio of H- to L-subunits in ferritin can vary widely. Ferritin H- and L-subunits are mapped on chromosomes 11q23 and 19q13.3, respectively, and both have multiple pseudogenes [1]. H-ferritin plays a major role in the rapid detoxification of iron, while the L-subunit is involved in nucleation, mineralization and long-term storage of iron [10].

**Figure 2:**
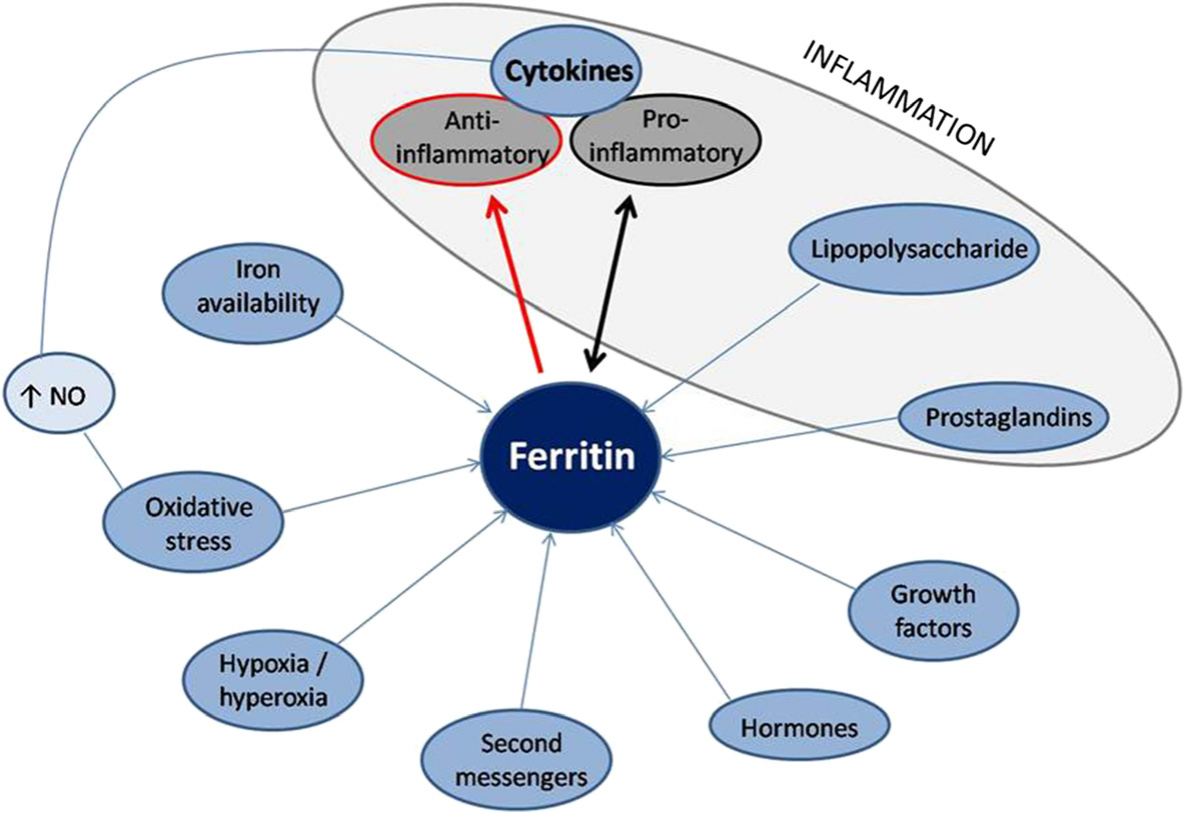
**Control of ferritin expression.** The expression of ferritin is regulated at both the transcriptional and post-transcriptional levels by iron, cytokine release, chemokine production, lipopolysaccharide, prostaglandins, hormones, growth factors, second messengers, hyperoxia and hypoxia, and oxidative stress [5]. Cytokines may also affect ferritin translation indirectly through their ability to induce nitric oxide synthase and, hence, increase nitric oxide (NO) (Figure 2) [11,12]. NO, in turn, causes inhibition of ferritin translation. Complex feedback mechanisms between ferritin and cytokines in the control of pro-inflammatory and anti-inflammatory mediators: cytokines can induce ferritin expression; otherwise, ferritin can induce the expression of pro- and anti-inflammatory cytokines.

Ferritin is a major intracellular iron storage protein in all organisms, and its structural properties are largely conserved through species (Figure 1). Each apoferritin (iron-free ferritin) shell comprises 24 subunits of two kinds: H-subunit and L-subunit. Depending on the tissue type and physiologic status of the cell, the ratio of H- to L-subunits in ferritin can vary widely, from a predominantly L-subunit rich ferritin in tissues such as liver and spleen, to H-subunit rich ferritin in the heart and kidneys [10]. The expression of ferritin is under delicate control (Figure 2). The amount of cytoplasmic ferritin is regulated by the translation of H- and L-ferritin mRNAs in response to an intracellular pool of “chelatable” or “labile” iron. In addition to iron, ferritin synthesis is regulated by cytokines at various levels (transcriptional, post-transcriptional and translational) during development, cellular differentiation, proliferation and inflammation [1]. Expression of ferritin is also regulated by oxidative stress, hormones (thyroid hormone), growth factors, second messengers, and hypoxia-ischemia and hyperoxia. Lipopolysaccharide (LPS - endotoxin), a component of the outer membrane of gram negative bacteria, elicits a variety of reactions that involve ferritin; in animal models the administration of LPS can increase ferritin expression. Also, cyclopentenone prostaglandins, which are involved in inflammatory and febrile responses as well as viral replication, induced L chain ferritin in human monocytes [1].

Hyperferritinemia is associated with several inflammatory conditions, such as sepsis, systemic inflammatory response syndrome (SIRS), multiorgan dysfunction syndrome (MODS), and MAS. In critically ill patients, hyperferritinemia is associated with the severity of the underlying disease [13-16]. In one study [14], very high levels of ferritin (>3,000 ng/ml) were associated with increased mortality in a dose response fashion.

The detailed secretory pathway of serum ferritin is not completely understood. Hepatocytes, macrophages and Kupffer cells secrete ferritin [2,17,18]. Serum ferritin is iron-poor and mainly consists of L-subunits [2]. So far, iron incorporation is the only L-ferritin function established by *in vitro* studies, but more recent studies showed that L-ferritin may have a stimulatory effect on cell proliferation, independent of iron availability. These findings suggest that L-ferritin may affect some cellular pathways that remain to be identified [19].

Moreover, there is still the paradox that circulating ferritin mainly consists of L-subunits, whereas most of the evidence supporting the existence of ferritin receptors indicates specificity for H-subunits [2].

The role of ferritin as a signaling molecule requires the presence of a specific receptor. Only the ferritin receptors expressed on hepatic cells bind both H- and L-ferritin, while those expressed on the other tissues are for the H-chain [20]. In an experimental murine model, the T-cell immunoglobulin and mucin domain (TIM)-2 was identified as a receptor for H-ferritin endocytosis in B and T cells, liver and kidney [21]. TIM-2 is a member of the T-cell TIM gene family, which is a family of cell surface molecules involved in the regulation of immune responses [17,21]. Recently, another cell surface receptor for ferritin, Scara5, was identified. Scara5 is a scavenger receptor that can bind various ligands, and, in contrast to TIM-2, it preferentially binds L-ferritin [22]. It is apparent that additional ferritin receptors may exist and have specific roles in different cell populations.

## Ferritin and immunity

### Ferritin as an immunosuppressant

H-ferritin has immunomodulatory effects, including suppression of the delayed type of hypersensitivity to induce anergy [23], suppression of antibody production by B lymphocytes [24], decreasing the phagocytosis by granulocytes [25], and regulating granulomonocytopoiesis [25]. Nevertheless, another ferritin-like molecule, a cloned human chimeric H-ferritin chain, PLIF (placenta immunomodulator ferritin), suppresses myelopoiesis and T cells, supporting the evidence that H-ferritin may have immunosuppressive functions [26]. The mechanisms underlying the inhibitory functions of H-ferritin are largely unknown, and they may include direct or indirect signaling *via* specific receptors for H-ferritin on lymphocytes [20] or the down-regulation of CD2, which acts as a cofactor for lymphocyte stimulation [27]. More recent data suggest that H-ferritin may suppress immune responses by its ability to induce production of the anti-inflammatory cytokine IL-10 in lymphocytes [28].

In addition to its suppressive effects on hematopoietic cell proliferation and differentiation, there is also evidence that H-ferritin plays an important role in chemokine receptor signaling and receptor-mediated cell migration. H-ferritin is a negative regulator of the CXC-chemokine receptor 4 (CXCR4). Thus, H-ferritin binding to CXCR4 impairs the signaling leading to the activation of mitogen-activated protein kinase (MAPK), a kinase that is known to play an important role in cell proliferation, differentiation and migration [29].

### Ferritin as a pro-inflammatory mediator

A novel role for extracellular ferritin as a pro-inflammatory signaling molecule in hepatic stellate cells has been proposed by Ruddell *et al.*[30]. Cells treated with ferritin activated a TIM-2-independent pathway comprising PI3 kinase phosphorylation, protein kinase C zeta activation and MAPK activation, ultimately culminating in activation of nuclear factor-κB (NF-κB). Activation of NF-κB in turn enhanced the expression of pro-inflammatory mediators, including IL-1β, inducible nitric oxide synthase and others. Of great relevance is the fact that this function was independent of the iron content of ferritin, suggesting that exogenous ferritin may assume roles entirely independent of its classic role as an iron binding protein. Moreover, this study showed that L-chain-rich tissue ferritin, and recombinant H- and L-ferritin, all initiated the activation of signaling pathways, which clearly suggests a role for serum ferritin (that is constituted mainly of L-ferritin subunits) as a pro-inflammatory mediator. Also, it was proposed that ferritin may play a role in an array of inflammatory/fibrogenic states associated with infection in organs, such as the heart, lungs, kidney and pancreas, all of which have cell types similar to hepatic stellate cells that mediate the fibrogenic response to injury [17,30].

A comprehensive analysis of the role of ferritin as a signaling molecule via TIM-2, Scara5 or via as yet unidentified receptors, will be of great interest and may lead to a better understanding of the precise role of circulating ferritin in inflammation.

### Ferritin in autoimmune diseases

Hyperferritinemia is known to be associated with autoimmune diseases, such as SLE, RA and MS [3-7], and also in serological antiphospholipid syndrome (APS) [8] (Table 1). The relevance of ferritin in autoimmune diseases is also supported by the finding of autoantibodies against ferritin in different autoimmune diseases: RA [31], giant cell arteritis and polymyalgia rheumatica [32] and Takayasu arteritis [33]. Yet, their importance remains to be established.

**Table 1:** Associations between hyperferritinemia and autoimmune diseases

	**Hyperferritinemia (%)**	**Described associations between hyperferritinemia and autoimmune diseases**
RA	4% [7]	✓ High concentrations of ferritin are found in synovial fluid and synovial cells of RA patients [5].
		✓ Significant correlations described between serum ferritin levels and disease activity by DAS28 score in RA patients [5].
MS	8% [6,7]	✓ Loss of ferritin binding is involved in, or is a consequence of, demyelination associated with MS [4].
		✓ Ferritin levels are significantly elevated in the serum and the cerebrospinal fluid only in chronic progressive active patients [4].
		✓ Hyperferritinemia is associated with male gender and a more progressive type of MS (that is, relapsing-progressive), whereas an inverse association was noted between the milder form of disease (relapsing-remitting) [6].
SLE	23% [7]	✓ Serum levels of ferritin during the more active stage of SLE exceeded those of RA patients and patients at less active stages of SLE [3].
		✓ Hyperferritinemia is associated with serositis and hematological manifestation [4].
		✓ ECLAM score is significantly higher in patients with hyperferritinemia [5].
		✓ Hyperferritinemia is associated with thrombocytopenia, lupus anticoagulant and anticardiolipin antibodies in SLE patients with active disease [5].
APS	Primary APS 8%	✓ In patients with APS syndrome, hyperferritinemia is associated with the presence of venous thrombotic events, cardiac, neurological and hematological manifestations [8].
	Secondary APS 9% [8]	

*APS* antiphospholipid syndrome, *DAS28* Disease Activity Score 28, *ECLAM* European Consensus Lupus Activity Measurement, *MS* multiple sclerosis, *RA* rheumatoid arthritis, *SLE* systemic lupus erythematosus.

The murine TIM gene family is linked to a locus that regulates airway hypersensitivity and the production of Th2 cytokines. Furthermore, in many of the animal autoimmune disease models in which a number of susceptibility loci have been identified, locus 11, which includes the TIM gene family, has been found to be related to susceptibility to autoimmunity [2,34,35]. Some polymorphisms in TIM genes are associated with immunity-related diseases, such as RA [34,35]. Additionally, it is known that TIM-2 is a negative regulator of the cells involved in the Th2 immune reaction [2,36,37]. The fact that ferritin acts as an immunosuppressant, together with the finding that TIM-2 is a specific receptor for ferritin, led Recalcati *et al.*[2] to propose that H-ferritin may have a role in autoimmunity. Different mechanisms involving H-ferritin/TIM-2 interactions can inhibit the H-ferritin-mediated suppression of immune cells. In turn, the impaired immunosuppression may favor the loss of tolerance and the development of autoimmune diseases [2].

Ferritin may also play a role in autoimmunity through its effects on CXCR4. As previously reported, H-ferritin is a negative regulator of CXCR4. This chemokine receptor is known to be significantly up-regulated in monocytes, neutrophils, B cell subsets and plasma cells in murine models of lupus nephritis. Moreover, the treatment of these mice with an antagonist of CXCR4 ameliorated end organ disease [38].

As described above, pro-inflammatory cytokines can induce ferritin expression; in turn, ferritin may induce the expression of pro-inflammatory cytokines. Moreover, ferritin induction of anti-inflammatory cytokines (IL-10) is an important mechanism underlying the immunosuppressive effects of ferritin. There seems, therefore, to be a complex interaction between ferritin and cytokines in the control of pro-inflammatory and anti-inflammatory mediators (Figure 2). So, ferritin can be either an immunosuppressive or a pro-inflammatory molecule. These opposing effects are probably dependent on the activation of different pathways, through different receptors, possibly employing different effectors (that is, L- versus H-ferritin), and maybe different contexts. In fact, this last idea resembles the two-hit hypothesis, for instance, *in vivo*, for the high levels of ferritin to be pathogenic it may require a second hit, like a pro-inflammatory environment, a specific infection or maybe a particular genetic background. Indeed, this may explain why in the case of hyperferritinemia-cataract syndrome there are high levels of ferritin without an inflammatory response.

MAS, AOSD, cAPS and septic shock are characterized by life-threatening hyperinflammation with multi-organ failure. Below we will review each one of these conditions in turn and Table 2 summarizes their clinical and laboratory features.

**Table 2:** Common clinical manifestations and laboratory abnormalities: MAS, AOSD, cAPS and septic shock

	**Septic shock**	**cAPS**	**AOSD**	**MAS**
Hyperferritinemia	+ [15,39]	[71%] [8]	[70 to 89%] [40,41]	[87 to 100%] [42]
Range of ferritin levels (ng/mL)*	21 to 2,210 [15]	250 to 2,875 [8]	223,6 to 54924 [43]	994 to 189,721 [44]
Hypercytokinemia	+ [45]	+ [46], [47]	+ [48]	+ [49-53]
Infection as a trigger	[100%] [54]	+ [46]	+ [41]	+ [55]
Fever	+ [54]	+ [56]	[82 to 100%] [41]	[78 to 94%] [42]
Multiorgan involvement	[100%] [54]	[100%] [46]	+ [41]	+ [14,55,57]
Hepatomegaly	Rare [14]	NR	[42%] [41]	[61 to 88%] [42]
Splenomegaly	Rare [14]	NR	[22 to 65%] [41]	[45 to 59%] [42]
Hemophagocytosis	+ [14]	NR	+ [3,40]	[81%] [42]
Thrombocytopenia	+ [14], [54]	[46%] [58]	-	[89%] [42]
Anemia	+ [54]	Hemolytic anemia [35%] [58]	[68%] [41]	[67 to 82%] [42]
Leukopenia	+ [14], [54]	NR	-	[39 to 56%] [42]
Neutropenia	+ [54]	NR	-	+ [14,55,57]
Neutrophilia	+ [54]	+ [56]	[81%] [41]	-
Macrophage activation	+ [14]	NR	+ [59]	+ [14,55,57]
Low/absent NK activity	+ [14]	NR	+ [60]	+ [14,55,57]
Sol. IL-2R >2,400 U/ml	+ [14]	NR	+ [48]	+ [14,55,57]
Abnormal liver function tests	+ [54]	+ [56]	[73%] [41]	[94%] [42]
HyperTG	+ [14]	NR	NR	[77 to 100%] [42]
Coagulopathy	+ [54]	DIC [15%] [58]	Rare [41]	+ [55]
Hypofibrinogenemia	+ [14], [54]	[15%] [58]	Rare [41]	[78 to 89%] [42]
ESR/CRP (↑ or ↓)	↑ [54]	↑ [46]	↑ [99%] [41]	ESR ↓ [79 to 92%] [42] CRP ↑ [61]

[%], percentage of association reported in the literature; **+**, positive association but not precise percentage reported; **-**, not associated; NR, no reported association.

*CRP* C reactive protein, *DIC* disseminated intravascular coagulation, *ESR* elevated sedimentation rate, *hyperTG* hypertriglyceridemia, sol. IL-2R, soluble IL-2 receptor.

* There is only our study on cAPS and it is a small cohort, and there are only a few studies on ferritin levels in sepsis, so the values of ferritin in these two conditions may be underestimated.

Table 2. All four conditions are life-threatening events in which an uncontrolled and immune response, triggered in most cases by infectious agents, leads to a severe hyperinflammation. There is evidence of hypercytokinemia and hyperferritinemia during the symptomatic period of the diseases. With the exception of the cAPS, for which there is no information in the literature, there is an impaired or absent function in natural killer (NK) and cytotoxic T cells.

## Clinical and laboratory features in mas, AOSD, cAPS and septic shock 

### Macrophage activation syndrome (MAS)

Hemophagocytic syndrome, also referred to as hemophagocytic lymphohistiocytosis (HLH), represents a severe hyperinflammatory condition triggered in most cases by infectious agents. Familial forms of HLH are due to mutations occurring either in the perforin gene or in genes important for the exocytosis of cytotoxic granules. Acquired forms of HLH are encountered in association with infections, autoimmune diseases, malignant diseases and acquired immune deficiency states (for example, after organ transplantation) [62].

An acquired form of HLH that occurs in autoimmune diseases is called MAS, and is most frequently seen complicating systemic juvenile idiopathic arthritis, but this syndrome has been increasingly reported in patients with SLE, AOSD, RA and less commonly in spondyloarthropathy and vasculitis [49]. MAS, like other forms of HLH, is characterized by prolonged fever, hepatosplenomegaly, cytopenias, high levels of ferritin, triglycerides, transaminases and bilirubin, and low fibrinogen [62]. Hemophagocytosis is often absent at the disease onset but is usually found with the progression of the disease. The soluble IL-2 receptor is a valuable disease marker because of consistently increased levels during active HLH [55]. MAS is a prototype of a major immune system activation characterized by enormous levels of ferritin and severe hypercytokinemia: IL-1β, IFN-γ, TNF-α, IL-10, IL-6, IL-18, IL-2 and IL-12 [49].

The pathogenesis is poorly understood, but in both genetic as well as in the acquired cases there is an impaired or absent function in natural killer (NK) and cytotoxic T cells [55,63].

Despite the close relationship of MAS with other forms of HLH, there are important clinical, laboratory and therapeutic differences that inclusively lead to a proposal of modified criteria for MAS [64]. In contrast to other forms of HLH, in MAS, cytopenias may be less severe initially, severe cardiac impairment appears to be common and coagulopathy is more pronounced, the C-reactive protein tend to be higher and when the cytokine profile is compared, the pro-inflammatory IL-β is elevated and the concentrations of IL-6 and TNF-α tend to be higher [61]. Also, the response to treatment is different and most of the MAS cases respond to less aggressive therapy than do the genetic forms of HLH [55].

### Adult onset Still’s disease (AOSD)

AOSD is a systemic inflammatory disorder with unknown etiology, but it is hypothesized that it may be a reactive syndrome where various infectious agents may act as disease triggers in a genetically predisposed host [65]. It is characterized by fever, arthritis and a typical skin rash (non-pruritic, salmon-pink macular lesions on the trunk and extremities) correlating with diurnal fevers. Important laboratory findings include leukocytosis (predominantly neutrophils) and high levels of ferritin [40,48]. Elevated serum ferritin levels were seen in 89% of these patients in some series, nearly half of whom had levels greater than five times normal [40]. Similarly to MAS, macrophage activation may play an important role in hyperferritinemia as well as in the pathogenesis of AOSD [59]. Heightened soluble IL-2 receptor levels, a marker of T cell activation, were also reported in two distinct studies of AOSD patients, serving as a potential marker of disease activity [66,67]. Furthermore, reactive hemophagocytic syndrome is not uncommon in AOSD [3,40]. Recent studies revealed a pivotal role of several pro-inflammatory cytokines on AOSD, such as IL-1, IL-6, IL-8, TNF-α and IL-18 in disease pathogenesis. There are controversial statements concerning the importance of IL-18 in distinguishing AOSD from other diagnoses [68,69]. NK T cells are numerically and functionally deficient in AOSD, similar to those observed in SLE, RA and MAS [60].

### Catastrophic antiphospholipid syndrome (cAPS)

The catastrophic variant of the APS syndrome is characterized by clinical evidence of multiple organ involvement developing over a very short period of time, histopathological evidence of multiple small vessel occlusions and laboratory confirmation of the presence of antiphospholipid antibodies (aPL), usually in high titer. Approximately 55% of cAPS cases are associated with a known trigger, such as infection or trauma [47,58,70]. We found that hyperferritinemia was strongly allied to the catastrophic variant of APS, present among 71% of cAPS patients with very high levels of ferritin (>1,000 ng/ml) determined in 36% of patients (although the cohort was small so the ferritin levels may be underestimated) [8]. Although patients with cAPS represent less than 1% of all APS patients, this complication can be life-threatening with a significantly increased mortality rate [46,56,58]. The mechanisms of cAPS are not clearly understood. The clinical manifestations of cAPS probably depend both on the organs affected by the thrombotic events, the extent of the thromboses and on the manifestations of the SIRS [47]. It is assumed that this multisystem inflammatory syndrome is caused by cytokine activation, although actual measurements of cytokine levels in very ill patients with cAPS have not been undertaken. Cytokines involved include TNF-α, IL-1, IL-6, IL-18 and macrophage-migration inhibitory factor [46].

### Septic shock 

Septic shock is thought to be a SIRS that is activated by invasive infection. The definition of septic shock includes sepsis-induced hypotension despite adequate fluid resuscitation, along with the presence of organ perfusion abnormalities, and ultimately cell dysfunction [54]. Hyperferritinemia is also known to be associated with sepsis [39]. Children with septic shock have hyperferritinemia and the levels of ferritin are associated with poor outcome [15]. Pro- and anti-inflammatory hypercytokinemia play a pivotal role in the pathophysiology of sepsis contributing to the dysregulation of the host immune system, inflammatory response and coagulation system [45,71,72]. Decreased NK cell activity is found in septic patients and is a predictor of neonatal sepsis [14].

### Efficacy of similar treatment modalities for the four clinical conditions 

Believing that ferritin may be pathogenic in these diseases, it would be expected that its decrease would ameliorate the clinical condition of the patients with these diseases. In fact, previously, hyperferritinemia in sepsis/MODS/MAS was successfully treated with plasma exchange, intravenous immunoglobulin (IVIG) and methylprednisone [16]. Indeed, these therapies were effective modalities, individually or in combination, in the four clinical conditions as described above (summarized in Table 3).

**Table 3:** The effectiveness of common treatment modalities: MAS, AOSD, septic shock and cAPS

	**Corticosteroids**	**IVIG**	**Blood purification/Plasma exchange**	**Others**
**MAS**	+++ [55]	++ [55]	++ [16,73-75]	Cyclosporine A [55]
**AOSD**	+++ [41,65]	++ [41,76]	+ [59,77,78]	DMARDs [41,65]; Anti-IL-6 [41,48]; Anti-IL-1 [41,48]
**cAPS**	+++ [46]	+++ [46,79,80]	+++ [46,81]	Anticoagulation [46,70]
**Septic shock**	+/− [54,82,83]	+/− [84]	++ [85-88]	Antibiotics [54]
**Rationale**	Anti-inflammatory effects of corticosteroids rely on their ability to repress the activity of immunomodulatory transcriptor factors like NF-κB and activator protein-1 [89].	Direct antitoxic effects, as well as indirect immunomodulatory mechanisms of IVIG has been described in the literature [84].	The overall concept of blood purification is to attenuate the overwhelming systemic overflow of pro- and anti-inflammatory mediators and to restore a broad-based humoral homeostasis [90].	
		IVIG probably acts by cytokine- and pathogen-specific antibodies [55,91].		
	They are cytotoxic for lymphocytes and inhibit expression of cytokines and differentiation of dendritic cells [55].	IVIG prevents release of pro-inflammatory cytokines in human monocytic cells stimulated with procalcitonin [92].	It is an extracorporeal blood purification technique designed to remove various toxic and inflammatory mediators and to replenish essential compounds via the replacement plasma [16].	

**+++** first line treatment recommended in international literature, **++** recommended treatment based in series cases reported in the literature, **+** treatment used in clinical practice described in case reports, **+/−** controversial use in clinical practice. *AOSD* adult onset Still’s disease, *cAPS* catastrophic antiphospholipid syndrome, *DMARDs* disease-modifying antirheumatic drugs, *IVIG* intravenous immunoglobulin, *MAS* macrophage activation syndrome, *NF-κB* nuclear factor kappaB, *NR* not reported.

Corticosteroids harbor anti-inflammatory effects that rely on their ability to repress the activity of immunomodulatory transcriptor factors, such as NF-κB and activator protein (AP)-1 [89]. They are cytotoxic for lymphocytes and inhibit expression of cytokines and differentiation of dendritic cells [55]. For patients with MAS, an acquired form of HLH, it has been proven that a less cytotoxic approach is effective, in contrast to the genetic forms of HLH in which an aggressive chemoimmune therapy is required [16]. In MAS high-dose corticosteroids is often used with good response [55]. Also in AOSD, corticosteroid therapy is effective in approximately two-thirds of patients [41,48]. Furthermore, in cAPS, corticosteroids may be considered in all patients unless an absolute contraindication exists; of course, that particular caution should be exercised in patients with infection [58]. Although some studies showed promising results with the use of corticosteroids in the treatment of sepsis and septic shock, larger studies and meta-analyses have failed to reproduce these effects. Hence, the utilization of corticosteroids in the treatment of sepsis remains controversial [82].

IVIG therapy is beneficial in a large number of autoantibody-mediated or self-reactive T cell-associated autoimmune diseases [55,91]. Direct antitoxic effects, as well as the indirect immunomodulatory mechanisms of IVIG are the basis for the rationale to use these substances in life-threatening infections and hyperinflammatory states [84]. IVIG probably acts by cytokine- and pathogen-specific antibodies, possibly including antibodies to ferritin [55,91]. Moreover, IVIG prevents the release of pro-inflammatory cytokines in human monocytic cells stimulated with procalcitonin [92]. IVIG is an important modality in the treatment of MAS [93], AOSD [65,76] and cAPS [79,80]. IVIG is not recommended in adult patients with septic shock, mainly due to the risk-benefit ratio and cost effectiveness [84].

Systemic inflammatory response is responsible for an important immunologic disturbance with the release into the bloodstream of numerous inflammatory mediators, such as cytokines, chemokines, complement components, platelet-activating factor, leukotrienes, thromboxanes and kinins. The overall concept of blood purification is, therefore, to attenuate this overwhelming systemic overflow of pro- and anti-inflammatory mediators released at the early phase of sepsis and to restore a broad-based humoral homeostasis in order to improve outcome [90]. Plasma exchange is an extracorporeal blood purification technique designed to remove various toxic and inflammatory mediators and to replenish essential compounds via the replacement plasma, which is known also to decrease ferritin levels [16]. It is a successful therapy in all four clinical conditions discussed, although in the case of the AOSD, there are only anecdotal cases [59,73-75,77,78,81,85-88].

On the other hand, there are also differences in the treatment of these conditions, for instance, Cyclosporin A, as part of the HLH-94 protocol, has been proven to be effective for maintaining remission in genetic HLH and for children with MAS [55], but its results in AOSD are modest [65]. As well, in cAPS the anticoagulation is one of the major therapies and is not indicated in the other conditions.

## Discussion

### The hyperferritinemic syndrome

The four conditions: MAS, AOSD, cAPS and septic shock share similar clinical signs, symptoms and laboratory parameters (summarized in Table 2). Additionally, they respond to similar modes of therapies (Table 3). Clinically, it is difficult to distinguish between these conditions; in fact, it was previously proposed that severe sepsis, SIRS and MAS could be considered intermediate phenotypes of the same inflammatory process, a spectrum of molecular abnormalities affecting target cells killed by cytotoxic T cells and NK cells [14]. Moreover, the overlap between MAS, cAPS and sepsis has been previously reported [94,95].

Information is emerging about the biological relevance of ferritin. Ferritin is known to be a pro-inflammatory mediator inducing expression of inflammatory molecules [30]. Yet it has opposing actions as a pro-inflammatory and as an immunosuppressant.

We believe that the very high ferritin levels in these clinical conditions are not just the product of the inflammation but rather may have a pathogenic role. Possibly, in an inflammatory environment, as observed in these diseases, the huge levels of ferritin may be involved in some sort of a loop mechanism where ferritin’s inflammatory proprieties are exacerbated, leading to an extreme expression of additional inflammatory mediators that are characteristic in the cytokine storm.

The good response to treatment with methylprednisolone, plasma exchange and IVIG supports a common pathogenic mechanism, and ferritin may be the link between them. It was previously shown that ferritin levels decreased gradually after each plasma exchange session [16]. Furthermore, IVIG may be relevant not only because antibodies against ferritin may be present, but it may also prevent the release of pro-inflammatory cytokines [92]. It is also very interesting to realize that the inhibition of the cytokines that play a central role in AOSD (IL-1 and IL-6) is an effective treatment, since they are the same cytokines known to induce ferritin expression [48]. Macrophages seem to play a major role in these four conditions. In fact, they are responsible for the production of cytokines and also appear to be of utmost importance in the production and secretion of serum ferritin.

However, not all the patients with these clinical conditions have hyperferritinemia; in fact, in about 10% of the AOSD patients the ferritin levels are normal [40]. Perhaps in this subgroup of patients the disease has a different etiology with a different pathogenesis. On the other hand, there are other diseases characterized by high levels of ferritin, such as hyperferritinemia-cataract syndrome that do not have an inflammatory response. Furthermore, the genetic forms of HLH that share clinical similarities with the four diseases discussed also have several important differences in the clinical, laboratory and, mainly, treatment response, which may suggest a distinct pathogenic features. Another clinical condition resembles these four that we have described, induced by the administration of an anti-CD28 monoclonal antibody. It led to a pro-inflammatory cytokine storm with multiorgan failure that responded to treatment with corticosteroids and hemodiafiltration with high dialysate rates and fresh frozen plasma. We may speculate that in this condition ferritin was also elevated, but it was not measured [96].

Taking this all together, we suggest that the four conditions: MAS, AOSD, cAPS and septic shock, which share common clinical and pathogenic features, should be included under a common syndrome named “Hyperferritinemic Syndrome”.

This concept of hyperferritinemia as a major contributor in the pathogenesis of these conditions may be extremely important in considering more targeted therapy. It is to be hoped that busy clinicians may appreciate the value of ferritin measurements when managing critically ill patients and that these assays may be useful in guiding therapy and predicting prognosis.

Further studies are required to understand the possible pathogenic role of ferritin in these conditions. There are many unsolved questions in this issue, such as why and how the serum ferritin is elevated, what is the composition of ferritin in the different diseases, and whether there are more receptors for ferritin and how ferritin interacts with them.

## Summary

● There is increasing evidence that circulating ferritin levels may not only reflect an acute phase response but may play a critical role in inflammation.

● MAS, AOSD, cAPS and septic shock are associated with very high levels of ferritin.

● These disorders share similar clinical and laboratory presentations and respond to similar treatments, suggesting that hyperferritinemia may be involved in a common pathogenic mechanism.

● We hypothesize that the huge levels of ferritin seen in these four clinical conditions are not just a secondary product of the inflammatory process, but rather, they are part of the pathogenic mechanism.

● We propose to include these four disorders under a single nomenclature: “The Hyperferritinemic Syndrome”.

## Abbreviations

AOSD: Adult onset Still’s disease; AP: Activator protein; aPL: Antiphospholipid antibodies; APS: Antiphospholipid syndrome; ARDS: Acute respiratory distress syndrome; cAPS: Catastrophic antiphospholipid syndrome; CXCR4: CXC-chemokine receptor 4; CXCL12: CXC chemokine ligand 12; DAS28: Disease activity score 28; DMARDs: Disease-modifying antirheumatic drugs; HLH: Hemophagocytic lymphohistiocytosis; IFN-γ: Interferon-γ; IL: Interleukin; IVIG: Intravenous immunoglobulin; LPS: Lipopolysaccharide; MAPK: Mitogen-activated protein kinase; MAS: Macrophage activation syndrome; MODS: Multiorganic dysfunction syndrome; MS: Multiple sclerosis; NF-kB: Nuclear factor-kB; NK: Natural kill; NO: Nitric oxide; PLIF: Placenta immunomodulator ferritin; RA: Rheumatoid arthritis; SIRS: Systemic Inflammatory Response Syndrome; SLE: Systemic lupus erythematosus; Th: T helper; TIM: T cell immunoglobulin and mucin-domain; TNF-α: Tumor necrosis factor alpha.

## Competing interests

The authors declare that they have no competing interests.

## Authors’ contributions

CR reviewed the literature and wrote the manuscript. GZ-G has experience in clinical research in ferritin and autoimmunity, and reviewed the manuscript. EGM-H has experience in basic research in ferritin and reviewed the manuscript. DPD’C contributed to the main idea and reviewed the manuscript. YS was responsible for the crystallization of the main idea and restructured the manuscript. All authors read and approved the final manuscript.

## Authors’ information

Cristina Rosário, MD, is a physician (Internist) in a public hospital and has experience with several autoimmune diseases as well as with patients with severe infections. She also did *in vivo* and *in vitro* research projects on ferritin and its implications in autoimmune and inflammatory diseases during her fellowship at the Zabludowicz Center for Autoimmune Diseases.

Gisele Zandman-Goddard, MD, is a head of the Department of Medicine and has experience with autoimmune diseases and has worked in several projects of basic research on ferritin and its relevance to autoimmune diseases.

Esther G. Meyron-Holtz, PhD, works on basic research with ferritin.

David P D’Cruz, MD, is head of the Department of Autoimmune Diseases, St Thomas Hospital London, U.K. He has experience with cAPS, vasculitides and other inflammatory autoimmune diseases.

Yehuda Shoenfeld, MD, is head of a center for autoimmune diseases. He has published extensively on autoimmunity and pathogenic factors, as well as on ferritin. Recently, he has coordinated scientific projects on basic research in ferritin and its implications in autoimmune and inflammatory diseases.
